# Dual Saturation in Soil Carbon Sequestration: Biophysical Limits and the Operational Capacity of Farmers

**DOI:** 10.1002/gch2.70118

**Published:** 2026-06-14

**Authors:** Moritz Von Cossel

**Affiliations:** ^1^ Biobased Resources in the Bioeconomy (340b) Institute of Crop Science University of Hohenheim Stuttgart Germany

**Keywords:** agricultural productivity, agri‐environmental policy, carbon farming, carbon sequestration, climate change mitigation, ecosystem, ecosystem services, environmental science, soil carbon, soil fertility

## Abstract

Global soils can store approximately 1500 Pg C in the upper meter and about 2344 Pg C within the upper 3 m, making soil organic carbon (SOC) the largest actively managed terrestrial carbon reservoir. Beyond its climate mitigation role, SOC underpins essential ecosystem services—including soil fertility, water regulation, and ecosystem stability—that support resilient agricultural landscapes and the long‐term reliability of food production systems. However, discussions of soil carbon sequestration often focus on biophysical potential while overlooking the human and institutional systems that determine whether this potential can be realized. This Perspective therefore introduces the concept of “dual saturation”, arguing that the global potential for soil carbon sequestration is constrained not only by biophysical limits of soil carbon stabilization but also by the operational capacity of farmers to implement complex ecological management practices. Biophysical saturation represents the desired restoration endpoint; operational saturation represents a systemic failure condition. The two are mutually exclusive in practice—and together constitute a self‐defeating state, since operational saturation prevents soils from ever approaching their biophysical ceiling. Drawing on evidence from agri‐environmental policy research and carbon‐farming initiatives, the article argues that administrative complexity, regulatory instability, and documentation requirements can reduce participation in soil carbon‐enhancing programs despite available incentives. Supporting farmers’ operational capacity through stable and low‐friction policy environments thus emerges as an essential enabling condition for large‐scale soil carbon restoration and for safeguarding the planetary health functions of agricultural landscapes.

## Graphical abstract

This Perspective introduces a dual saturation framework showing that soil carbon sequestration is constrained not only by biophysical limits, but also by social–institutional barriers. Policy complexity, documentation burdens, and operational saturation can restrict farmer adoption and create a mitigation gap, highlighting the importance of stable, transparent, and operationally feasible policy design for long‐term climate mitigation.

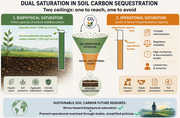

## 1 Introduction: The Stock, the Debt, and the Vulnerability

To understand the stakes of agricultural transition, one must view terrestrial soils within the broader global carbon budget. The atmospheric pool currently holds approximately 895 Pg C, while terrestrial vegetation stores roughly 560 Pg C [1]. These are dwarfed by the oceanic reservoir, which holds approximately 38000 Pg C [1]. While the marine pool is orders of magnitude larger, its deep‐ocean components remain largely beyond the reach of direct human management. Blue carbon ecosystems such as mangroves and seagrasses can sequester carbon rapidly, often at rates far exceeding those observed in terrestrial ecosystems [2, 3]. While blue carbon ecosystems offer locally important sequestration opportunities, their global spatial extent remains too limited to serve as a primary climate lever. Agricultural soils, by contrast, cover nearly half of the global ice‐free land surface and are directly shaped by the daily decisions of land managers worldwide, making them the most practically accessible reservoir for large‐scale carbon accumulation.

**FIGURE 1:**
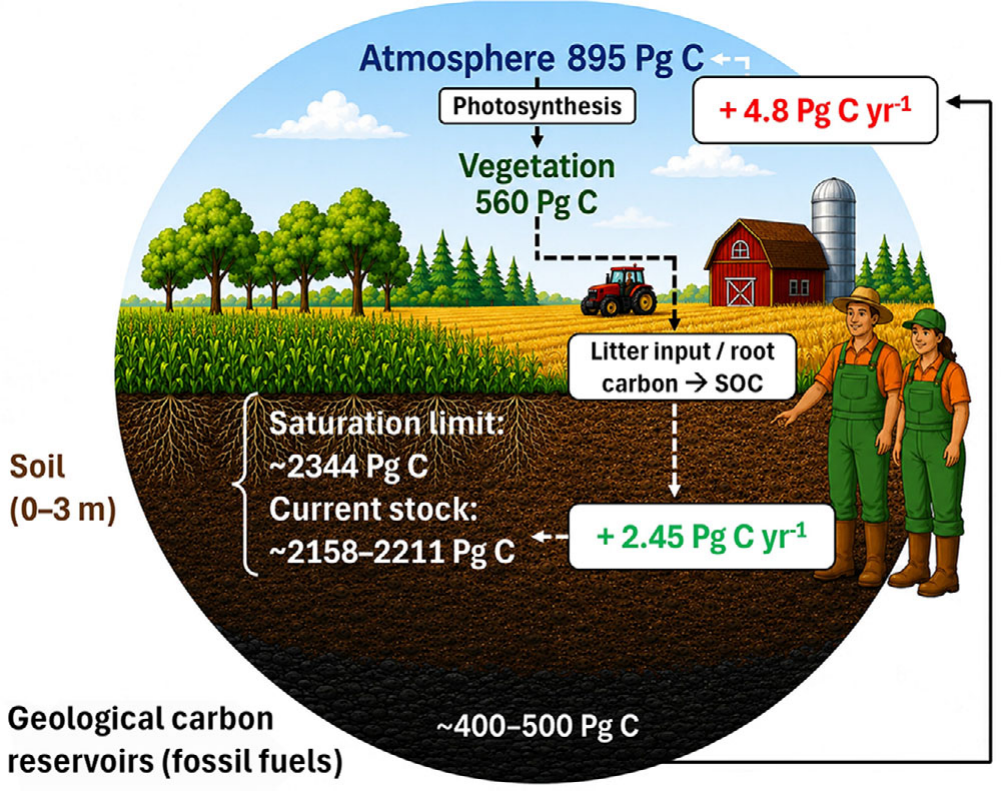
Global carbon reservoirs and flux potentials. The atmospheric pool (895 Pg C) is currently increasing at a rate of approximately 4.8 Pg C yr^−1^ [1]. While the terrestrial vegetation (approx. 560 Pg C) remains a dynamic but relatively stable reservoir, the maximum global soil organic carbon (SOC) stock—estimated at 2344 Pg C down to a depth of 3 m—represents the theoretical biophysical saturation ceiling of the largest manageable terrestrial carbon pool [5]. Current global SOC stocks are estimated at approximately 2158–2211 Pg C to this depth, reflecting a historical carbon debt of 133–186 Pg C accumulated through land use change since pre‐agricultural times [6]. The theoretical technical sequestration potential to "refill" depleted agricultural soils is estimated at 2.45 Pg C yr^−1^ [7], highlighting the soil's role as a critical, yet operationally sensitive, lever for climate mitigation. The solid arrow indicates the transfer of fossil carbon from geological reservoirs to the atmosphere, whereas the dashed arrows represent the potential biological pathway of atmospheric carbon uptake via vegetation (photosynthesis) and its transfer to soil organic carbon through plant inputs (e.g., litter and root‐derived carbon). Natural return fluxes of carbon from vegetation and soils to the atmosphere (e.g., respiration and mineralization) are not shown for clarity. Global proven fossil fuel reserves (i.e., economically recoverable fossil carbon stocks) are estimated at ∼400–500 Pg C, exceeding the remaining carbon budget for 1.5°C warming [8]. AI use disclosure: This figure was created as a composite using ChatGPT (OpenAI, GPT‐4o, 2025/2026), Microsoft PowerPoint, and Paint.NET (getpaint.net). The background landscape illustration and selected labels were AI‐generated; additional text elements, quantitative values, arrows, and text boxes were added or modified by the author in PowerPoint; image refinements were made using Paint.NET. The scientific content and all quantitative information are based exclusively on the reviewed literature. AI‐generated elements serve a purely illustrative and explanatory function and do not represent primary research data.

Managing this reservoir is not just a climate strategy, but a core component of the biodiversity‐water‐food‐health nexus. Recent assessments indicate that while most land‐based climate responses have broadly positive impacts across this nexus, their success depends on rapid, sustained mitigation and societal engagement [4]. In contrast, terrestrial agricultural land currently covers approximately 40% of the global ice‐free land surface, making agricultural soils one of the largest actively managed components of the terrestrial carbon cycle. This massive spatial footprint makes the global soil organic carbon (SOC) stock—estimated at 1500 Pg C in the top meter and 2344 Pg C down to 3 m (Figure 1) [5]—the most significant “manageable” lever for climate mitigation.

Soil organic carbon is therefore not only a climate mitigation resource but also a foundational component of planetary health [9, 10, 11]. By regulating soil fertility, water retention, and ecosystem stability, SOC supports the resilience of agricultural landscapes and the long‐term reliability of food production systems upon which human societies depend [12]. Declining soil carbon stocks have been associated with reduced agricultural productivity, increased vulnerability to climate extremes, and degradation of ecosystem services [10, 12]. Consequently, strategies that restore and stabilize SOC contribute not only to climate mitigation but also to the ecological foundations of human health and wellbeing within the Earth system.

It is crucial to distinguish this current stock from future sequestration potential. The 2344 Pg C baseline represents a reservoir that is currently highly vulnerable; unsustainable agricultural practices accelerate mineralization, potentially releasing portions of this pool back into the atmosphere. This “existing stock” is not a static bank, but a dynamic system currently in a state of net loss in many regions. Historical land use has already resulted in an estimated ‘carbon debt’ representing the depletion of soil organic carbon relative to pre‐agricultural conditions—conservatively estimated at 50–133 Pg C [13],  though more recent assessments suggest this debt likely falls within the range of 133–186 Pg C [6]. This loss defines a substantial opportunity for climate mitigation through the restoration of soil carbon stocks. By adopting regenerative management to “refill” this debt, global soils offer a technical sequestration potential of approximately 2.45 Pg C per year (range 1.45–3.44 Pg C yr^−1^) through the gradual rebuilding of depleted SOC pools [7]. However, the success of this sequestration is not simply a matter of adding new carbon, but of stabilizing the approximately 2344 Pg C already stored in global soils, whose persistence depends on soil structure, mineral associations, and land management. Unlike inaccessible marine sediments, the upper layers of the global soil profile are directly influenced by daily agricultural decisions, making SOC a uniquely accessible tool for carbon accumulation—provided the land manager is enabled to act. However, the ability of farmers to implement SOC‐enhancing management practices is not determined solely by biophysical knowledge or economic incentives. Increasing evidence suggests that administrative and regulatory complexity can significantly influence the adoption of environmentally beneficial farming practices. Studies across European agricultural policy frameworks have shown that perceived bureaucratic burden, including documentation, monitoring, and compliance reporting, strongly shapes farmers' willingness to participate in agri‐environmental programs [14, 15]. Similar concerns have been raised in the context of biodiversity conservation schemes and emerging soil carbon programs, where monitoring and verification requirements may increase transaction costs and reduce participation despite the availability of financial incentives [16, 17].

These findings highlight an often‐overlooked constraint in climate mitigation strategies based on agricultural soils: the operational capacity of farmers themselves. If the administrative burden associated with ecological management becomes excessive, the practical implementation of otherwise effective carbon sequestration strategies may be constrained not only by soil biogeochemistry but also by the institutional and administrative conditions under which farmers operate. This Perspective introduces the concept of dual saturation–two distinct ceilings that together constrain the realization of soil carbon sequestration potential: a biophysical ceiling to be actively pursued, and an operational ceiling defined by the implementation capacity of land managers, to be actively prevented.

While the global carbon stocks discussed above provide the broader climate context, the policy analysis presented in this Perspective primarily refers to agricultural policy environments in Europe, particularly within the framework of the European Union's Common Agricultural Policy (CAP). These policy systems provide one of the most extensive examples of large‐scale agri‐environmental governance and therefore offer a relevant case for examining how administrative complexity can influence the implementation of soil‐carbon‐enhancing practices.

## 2 The Biological Ceiling: The Knowledge Gap and Stoichiometric Constraints

Among the various strategies proposed to enhance soil carbon sequestration, biologically based farming systems—such as diversified crop rotations, cover cropping, and reduced disturbance—often require greater management complexity and observational effort than simplified input‐based systems [18, 19]. These systems can therefore be particularly sensitive to institutional instability or administrative burden, as their successful implementation depends on long‐term management planning and adaptive field‐level decision making [20, 21]. Historical SOC depletion in agricultural soils has been driven not only by nutrient imbalances but also by soil disturbance, loss of vegetation cover, degradation of soil structure, and simplification of cropping systems, which collectively reduce the capacity of soils to stabilize organic matter [22].

Biological sequestration in soils is often discussed in relation to carbon saturation concepts. At the same time, soil carbon dynamics are shaped by multiple ecological mechanisms beyond mineral surface saturation, including soil aggregation processes, particulate organic matter inputs, vegetation cover, and reduced disturbance practices. As Jobbágy and Jackson [5] showed, global soils currently store approximately 2344 Pg C down to a depth of 3 m, forming the largest terrestrial carbon reservoir directly influenced by land management decisions, although specific global estimates vary depending on depth and database used [23]. Soils act as sponges; once mineral surfaces are saturated, the rate of additional sequestration drops toward a new equilibrium. Furthermore, the stabilization of soil organic carbon is influenced by nutrient stoichiometry and microbial processing, although these relationships are not governed by a single fixed elemental ratio. Soil organic matter C:N ratios vary widely depending on soil mineralogy, vegetation inputs, and land management practices [24]. Carbon stabilization occurs through multiple mechanisms, including mineral association, particulate organic matter formation, aggregate protection, and the maintenance of vegetative cover that sustains continuous organic inputs. Consequently, soil carbon sequestration initiatives generally aim to increase total SOC stocks rather than targeting specific C:N ratios. Nutrient availability nevertheless remains an important enabling factor for sustained carbon accumulation in agricultural soils. It is important to note that the processes discussed here primarily refer to mineral agricultural soils. Carbon dynamics in peatlands and other organic soils follow distinct stabilization mechanisms and are governed by different hydrological and ecological controls [25].

The resistance to transitioning toward diversified crop rotations that include legumes, grasses, and cover crops reveals an important economic and management barrier. Such systems can enhance soil organic matter levels compared with simplified rotations, but they also require more complex management and multi‐year planning [26, 27, 28]. In contrast, managing a biological nitrogen source through the integration of specific legumes such as alfalfa (*Medicago sativa*), faba beans (*Vicia faba*), or clover‐grass leys to satisfy the C:N ratio defined by Tipping et al. [24] involves multi‐year planning and deep observational labor (Table 1).

**TABLE 1:** The transition friction—mechanical vs. biological paradigms.

Feature	Mechanical paradigm (industrial)	Biological paradigm (regenerative)
Primary nitrogen source	Synthetic fertilizer (e.g., urea application)	Leguminous fixation (e.g., clover ley, faba, bean rotation) & organic matter
Predictability	High (direct linear yield response)	Variable (dependent on soil biology/weather)
Operational labor	Low (single tractor pass)	High (cover crop establishment, termination, monitoring)
Cognitive load	Low (standardized application rates)	High (site‐specific observational mastery)
Administrative burden	Low (minimal reporting)	High (complexity of “green” compliance)
Risk profile	Financial (input price volatility); long‐term soil degradation risk if management is simplified	Operational (biological failure/harvest loss)

While synthetic nitrogen can be applied via a single tractor pass, biological systems require a massive increase in cognitive and observational labor. Importantly, soil carbon sequestration efforts typically focus on increasing SOC stocks rather than achieving specific stoichiometric ratios, which can vary considerably depending on soil type, vegetation inputs, and management practices. When the legislative environment is unstable, the risk‐to‐reward ratio for this high‐knowledge approach becomes unfavorable, as farmers are unlikely to invest the mental energy required to master complex rotations if the underlying subsidy structure might shift before the soil reaches a new equilibrium [14, 15]. The discussion here primarily refers to intensively managed agricultural systems typical of industrialized farming regions, where synthetic nitrogen inputs have historically played a central role in crop production [27, 29].

From a management perspective, soil carbon sequestration is closely linked with broader soil health processes, including nutrient cycling, water retention, and biological diversity [30]. These interacting components form a coupled system in which improvements in soil carbon often coincide with enhanced moisture regulation, nutrient availability, and ecological resilience.

In addition to management practices such as diversified rotations and reduced disturbance, technological interventions, including organic amendments and biochar applications, have also been proposed as strategies to enhance soil carbon stabilization [31, 32]. However, the successful implementation of these strategies ultimately depends on the management decisions and operational capacities of farmers within the institutional environments in which they operate.

## 3 The Human Ceiling: Beyond Legislative Periods

Translating soil carbon sequestration potential into realized climate mitigation requires more than understanding soil biogeochemistry. It also depends on the human systems that govern land management decisions.

While soil science has traditionally focused on the biophysical constraints governing soil organic carbon stabilization, the realization of this potential ultimately depends on human decision‐making within institutional contexts. Farmers operate at the interface between ecological processes and policy frameworks, translating scientific knowledge into day‐to‐day management decisions. Consequently, the achievable level of SOC sequestration is not determined solely by soil biogeochemistry but by the interaction between ecological potential and both social and institutional constraints under which land managers operate. Understanding this interaction is therefore essential for translating theoretical sequestration potential into realized climate mitigation within agricultural social‐ecological systems [30, 33].

Within European agricultural policy frameworks such as the Common Agricultural Policy (CAP), farmers’ engagement with environmental management schemes is strongly shaped by the transaction costs associated with participation. Recent research on agri‐environmental policy adoption suggests that these transaction costs—including administrative burden, monitoring obligations, and documentation requirements—can significantly influence farmers' willingness to participate in environmental subsidy programs [14, 15]. Similar concerns have been raised for emerging carbon farming initiatives, where monitoring, reporting, and verification requirements may increase transaction costs and thereby limit farmer participation in soil carbon sequestration programs despite their climate mitigation potential [17].

**FIGURE 2:**
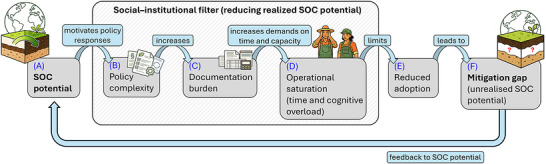
Social‐institutional filtering of soil organic carbon sequestration potential. The theoretical potential for soil organic carbon (SOC) sequestration (A) is mediated by social and institutional constraints that influence farmers’ capacity to implement carbon‐enhancing practices. Policy complexity and regulatory instability (B) increase the administrative workload associated with monitoring, reporting, and compliance requirements (C). This increased documentation burden raises demands on farmers’ time and cognitive capacity, contributing to operational saturation of land managers (D). As a consequence, adoption rates of SOC‐enhancing practices remain below their technical potential (E), producing a gap between achievable and realized climate mitigation (F). The arrows indicate the causal sequence linking these components. A feedback relationship illustrates how this mitigation gap reflects unrealized SOC potential. The diagram illustrates that human and institutional capacity can act as an important constraint influencing the extent to which biophysical SOC sequestration potential is realized. AI use disclosure: The icons used in this figure were generated with the assistance of ChatGPT (OpenAI, GPT‐4o, 2025/2026) and subsequently refined using Paint.NET (getpaint.net). The remaining visual elements, including the diagram layout, arrows, and all textual content, were created by the author using Microsoft PowerPoint. The conceptual framework and all scientific content were developed exclusively by the author. AI‐generated elements serve a purely illustrative function and do not represent primary research data.

One potentially overlooked constraint in climate mitigation may therefore be the operational saturation of land managers—the human dimension of the “dual saturation” framework. As illustrated in Figure 2, the theoretical biophysical potential for soil carbon sequestration is mediated by ecological, institutional, and societal constraints that together determine the extent to which this potential is realized in practice. Farmers are decision‐makers operating in high‐risk environments where the implementation of sequestration strategies is increasingly complex. Importantly, this constraint is self‐reinforcing: where the mitigation gap persists and atmospheric carbon concentrations continue to rise, the resulting acceleration of soil warming, drought frequency, and mineralisation rates may progressively erode the biophysical sequestration potential itself—creating a feedback from institutional failure to ecological constraint that is not captured by biophysical models alone (Figure 2).

While reviewers of agricultural policy often point to the success of past incentives—such as the rapid expansion of biogas in Germany under the Renewable Energy Sources Act (EEG), which demonstrated that clear economic signals can drive rapid agricultural transitions when best management practices are followed—these nonetheless represent ‘mechanical‐economic’ transitions characterised by high price certainty and standardised feedstocks, and their ecological outcomes depend critically on the agronomic care with which they are implemented. In contrast, increasing SOC requires a “biological‐observational” transition, which is currently hindered by what can be described as a “documentation tax”—the cumulative administrative burden associated with monitoring, reporting, and compliance requirements in environmental agricultural policy [34]. These examples highlight that adoption dynamics differ substantially between standardized technological transitions driven by clear economic incentives and biologically complex land‐management transitions that require long‐term observational and management effort.

For instance, analyses of cross‐compliance policies in European agriculture demonstrate that perceived administrative burden strongly shapes farmers' attitudes toward environmental policy instruments and can reduce their willingness to participate in subsidy‐linked environmental programs [14]. Similarly, survey‐based studies of result‐based agri‐environmental schemes in Germany identify bureaucratic complexity and reporting requirements as among the most important deterrents to participation, even when financial incentives are available [15]. These findings suggest that the effectiveness of environmental policy instruments is not determined solely by payment levels or ecological design, but also by the transaction costs imposed on farmers through monitoring, verification, and documentation requirements.

Empirical evidence now quantifies this friction. For example, research into the cultivation of industrial hemp—a high‐biomass crop often discussed in the context of carbon‐based bioeconomy systems [35, 36, 37]—demonstrates that administrative hurdles and regulatory compliance act as a primary barrier to adoption, effectively creating a “saturation” of the farmer's operational bandwidth [38]. Similarly, the transition to biodiversity‐friendly biomass systems, such as perennial wild plant mixtures, reveals that the ecological benefits of such systems are often neutralized by the cognitive and administrative load required to navigate their complex eligibility and reporting frameworks [39].

Comparable patterns have been documented across European biodiversity‐oriented agricultural programs. Reviews of result‐based conservation schemes highlight that many initiatives require extensive documentation, monitoring of ecological indicators, and detailed reporting on management practices such as mowing regimes, species composition, or field management timelines [17]. While these requirements are designed to ensure ecological outcomes, they can simultaneously increase the administrative workload for farmers and thereby limit participation rates.

This challenge is particularly relevant for soil‐carbon‐related initiatives. Reviews of soil carbon sequestration programs and emerging carbon farming schemes indicate that monitoring, reporting, and verification requirements represent one of the most significant practical barriers to farmer engagement in soil‐carbon initiatives [16]. Documenting practices, verifying carbon outcomes, and navigating program rules can increase transaction costs and reduce the attractiveness of participation, especially when expected financial returns remain uncertain.

This diverted time reduces the capacity for adaptive management required to respond to site‐specific soil conditions observable through crop performance, soil structure, moisture dynamics, and other field indicators. Every hour spent navigating regulatory flux—the periodic adjustment of eligibility criteria, reporting requirements, and compliance rules across successive policy cycles within the European Common Agricultural Policy—is an hour diverted from field‐based observation [40]. When reporting requirements become dominant in farm management routines, time available for field observation and adaptive decision‐making may decrease, potentially reducing what has been described as farmers' “situational awareness” [41]. Taken together, these findings reinforce the argument that administrative complexity can reduce the adoption of environmentally beneficial farming practices by shifting farmer effort from ecological management toward regulatory compliance.

While SOC requires 20–50 years to reach a new equilibrium [42], the current administrative environment forces farmers into a reactive, short‐term compliance mode. If participation in soil‐carbon or biodiversity schemes depends on extensive documentation and monitoring obligations, many farmers may rationally avoid entering such programs despite their ecological benefits. To achieve global sequestration goals, it is therefore important to recognize that protecting the farmer's mental and operational bandwidth may be as important for implementation as traditional agronomic constraints such as nitrogen or water availability.

## 4 Policy as a “Tragedy of the Horizon”

Applying Mark Carney's concept of the ‘Tragedy of the Horizon'—which describes the mismatch between long‐term climate risks and short‐term political and financial decision horizons—to soil science reveals a fundamental governance challenge [43]. The physical and microbial timeframes required to reach a new SOC equilibrium are measured in decades (20–50 years) [42], yet political cycles and current economic assessments [44] operate on significantly shorter horizons. This mismatch creates a “valuation gap” where the long‐term resilience of soil is not recognized as a capital asset. At the same time, policy stability must be balanced with periodic evaluation to prevent unintended land‐use outcomes, as illustrated by past policy experiences in bioenergy expansion.

To bridge this gap, I propose a move toward what I term Decadal‐Scale Governance—drawing on the global soil climate mitigation strategy outlined by Amelung et al. [30]—built on four pillars:

1. Long‐term regulatory horizons: agricultural policies must be decoupled from short‐term election cycles [45], providing farmers with 10‐to‐20‐year stability to realize the biological transitions necessary for SOC equilibrium.
2. Stoichiometric support: policies must incentivise the integration of diverse legume rotations and nitrogen‐fixing cover crops (e.g., winter vetch or crimson clover) to address what may be termed the ‘nitrogen bottleneck’—the limited availability of biologically fixed nitrogen relative to the nitrogen demand of sustained soil carbon accumulation [24, 46] by aligning carbon sequestration goals with the biological necessity of nitrogen inputs, while reducing national exposure to synthetic fertilizer price shocks [47].
3. Outcome‐based monitoring: where feasible, technological tools such as soil monitoring platforms or geospatial tracking may support verification while minimizing reporting complexity; however, monitoring systems must be carefully designed to avoid increasing the administrative burden that already limits participation in environmental programs [48, 49].
4. Social resilience: policy frameworks should consider the administrative and cognitive workload placed on farmers, recognizing that excessive regulatory complexity can undermine the effective management of biologically complex agricultural systems [50].

## 5 Conclusion: The Resilience of the Social‐Ecological System

The global soil carbon reservoir represents a substantial opportunity for climate mitigation. However, evidence from research on ecosystem services and agricultural systems suggests that ecological resilience cannot be sustained independently of the social systems that manage it. Soil health and farmer wellbeing are interconnected components of agricultural social–ecological systems, in which ecological processes, management decisions, and institutional conditions interact to shape long‐term system stability [11, 33].

Achieving soil‐based climate mitigation targets requires not only optimizing biophysical processes but also ensuring that farmers have the operational capacity to implement complex ecological management practices. Policy frameworks that reduce administrative friction and provide long‐term regulatory stability may therefore represent essential enabling conditions for large‐scale soil carbon restoration. Recognizing farmer wellbeing and institutional design as integral components of soil carbon dynamics could improve the effectiveness and scalability of climate mitigation strategies within agricultural systems. In this context, reducing operational constraints on farmers may support more adaptive management, enhance ecological outcomes, and contribute to more resilient food systems [4, 51].

It is worth reflecting on the normative asymmetry embedded in the dual saturation framework itself. Biophysical saturation represents the desired endpoint of soil carbon restoration—a condition to be actively pursued. Operational saturation represents a systemic failure condition—a point to be actively prevented. In this sense, dual saturation is ultimately a self‐defeating state: a diagnostic phantom rather than an achievable reality. If farmers reach operational saturation before soils approach biophysical saturation, the restoration goal recedes further from reach; and if soils were ever to approach biophysical saturation, it would only be because operational burdens had been successfully kept at bay. The two ceilings are mutually exclusive in practice. The policy implication is correspondingly asymmetric: strive toward one form of saturation, and deliberately keep the other as far from its limit as possible. Ultimately, the real mitigation potential depends not only on the carbon stored beneath our feet, but on the long‐term decisions of the farmers who manage this reservoir—and on whether the institutional environments in which they operate enable or obstruct those decisions, with implications not only for climate mitigation but also for the resilience of food systems and the broader determinants of human health.

## Author Contributions

Moritz von Cossel: conceptualization, methodology, formal analysis, investigation, visualization, writing – original draft, writing – review & editing.

## Artificial Intelligence Statement

Claude (Anthropic), ChatGPT (OpenAI), and Gemini (Google) were used as assistive tools during manuscript preparation. In all cases, the author independently defined the scientific content, conceptual framework, and arguments; AI tools supported phrasing refinement, clarity improvement, and consistency checking. For the figures and graphical abstract, AI‐based image generation tools assisted with graphical presentation of author‐defined content. All AI‐assisted outputs were critically reviewed and approved by the author, who bears full responsibility for the scientific integrity of this work.

## Funding

This study received no research funding.

## Conflicts of Interest

The authors declare no conflicts of interest.

## Data Availability

Data sharing not applicable to this article as no datasets were generated or analysed during the current study.
