# Correction to “Dual Saturation in Soil Carbon Sequestration: Biophysical Limits and the Operational Capacity of Farmers”

**DOI:** 10.1002/gch2.70152

**Published:** 2026-09-22

M. von Cossel, “Dual Saturation in Soil Carbon Sequestration: Biophysical Limits and the Operational Capacity of Farmers,” *Global Challenges* *10*, no. 6, (2026): e70118, https://doi.org/10.1002/gch2.70118.

In the abstract, the text “Global soils can store approximately 1500 Pg C in the upper meter and about 2344 Pg C within the upper 3 m, making soil organic carbon (SOC) the largest actively managed terrestrial carbon reservoir.” was incorrect. This should have read: “Global soils have been estimated to contain approximately 1500 Pg C in the upper meter and approximately 2344 Pg C within the upper 3 m, making SOC the largest terrestrial organic carbon reservoir substantially influenced by land management.”

In paragraph 2 of the “Introduction” section, the text “In contrast, terrestrial agricultural land currently covers approximately 40% of the global ice‐free land surface, making agricultural soils one of the largest actively managed components of the terrestrial carbon cycle. This massive spatial footprint makes the global soil organic carbon (SOC) stock, which is estimated at 1500 Pg C in the top meter and 2344 Pg C down to 3 m (Figure 1) [5], the most significant “manageable” lever for climate mitigation.” was incorrect. This should have read: “The massive spatial footprint makes the global soil organic carbon (SOC) stock, which is estimated at 1500 Pg C in the top meter and 2344 Pg C down to 3 m (Figure 1) [5], the most significant “manageable” lever for climate mitigation.”

In paragraph 3 of the “Introduction” section, the text “Declining soil carbon stocks have been associated with reduced agricultural productivity, increased vulnerability to climate extremes, and degradation of ecosystem services [10, 12].” was incorrect. This should have read: “Declining SOC stocks have been associated with reduced agricultural productivity, increased vulnerability to climate extremes, and degradation of ecosystem services [10, 12].”

In paragraph 4 of the “Introduction” section, the text “Historical land use has already resulted in an estimated “carbon debt” representing the depletion of soil organic carbon relative to pre‐agricultural conditions, conservatively estimated at 50–133 Pg C [13], though more recent assessments suggest this debt likely falls within the range of 133–186 Pg C [6].” was incorrect. This should have read: “Historical land use has already resulted in an estimated “carbon debt” representing the depletion of SOC relative to pre‐agricultural conditions, conservatively estimated at 55–78 Pg C [13], with more recent data‐driven modelling suggesting a global agricultural debt of approximately 116 Pg C for the upper 2 m of soil [6].”

Furthermore, in paragraph 4 of the “Introduction” section, the text “However, the success of this sequestration is not simply a matter of adding new carbon, but of stabilizing the approximately 2344 Pg C already stored in global soils, whose persistence depends on soil structure, mineral associations, and land management.” was incorrect. This should have read: “However, the success of this sequestration is not simply a matter of adding new carbon, but of stabilizing the approximately 2344 Pg C already stored in global soils (0–3 m), whose persistence depends on soil structure, mineral associations, and land management.”

In the caption of Figure 1, the text “While the terrestrial vegetation (approx. 560 Pg C) remains a dynamic but relatively stable reservoir, the maximum global soil organic carbon (SOC) stock–estimated at 2344 Pg C down to a depth of 3 m–represents the theoretical biophysical saturation ceiling of the largest manageable terrestrial carbon pool [5]. Current global SOC stocks are estimated at approximately 2158–2211 Pg C to this depth, reflecting a historical carbon debt of 133–186 Pg C accumulated through land use change since pre‐agricultural times [6].” was incorrect. This should have read: “While the terrestrial vegetation (approx. 560 Pg C) remains a dynamic but relatively stable reservoir, the global soil organic carbon (SOC) stock, which is estimated at approximately 2344 Pg C down to a depth of 3 m, represents the largest terrestrial organic carbon pool, substantial portions of which are directly or indirectly influenced by land use and land management [5]. The theoretical biophysical saturation ceiling of global SOC stocks remains unknown, because available global inventories estimate existing stocks rather than the maximum amount of carbon that soils could stabilize. Estimates of historical SOC losses range from approximately 55–78 Pg C for managed ecosystems [13] to approximately 116 Pg C for global agricultural soils to 2 m depth [6].”

In Figure 1, the text “Saturation limit: ∼2344 Pg C” and “Current stock: ∼2158–2211 Pg C” was incorrect. This should have read: “Current stock: ∼2344 Pg C”. The corrected version of Figure 1 is shown below.


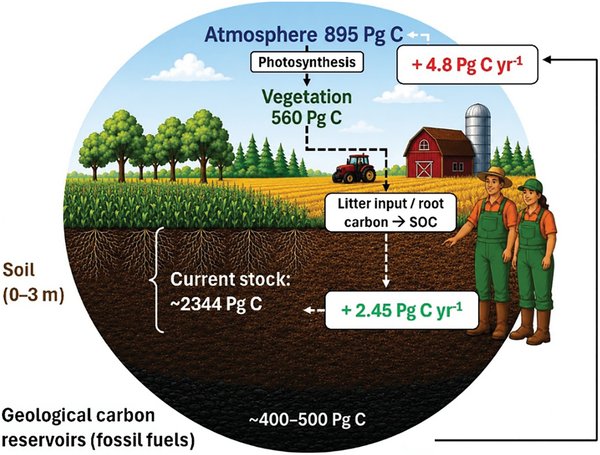


**Figure 1 (corrected)**.

In the Graphical Abstract, the text “Saturation limit (∼2344 Pg C to 3m)” and “Current estimated soil organic carbon content (∼2211 Pg C to 3 m)” was incorrect. This should have read: “Global SOC saturation level (unknown)” and “Current stock (0–3 m): ∼2344 Pg C (Jobbágy and Jackson, 2000)”. Further, the depiction of the green bar was adjusted to better represent the corrected interpretation. The corrected version of the graphical abstract is shown below.


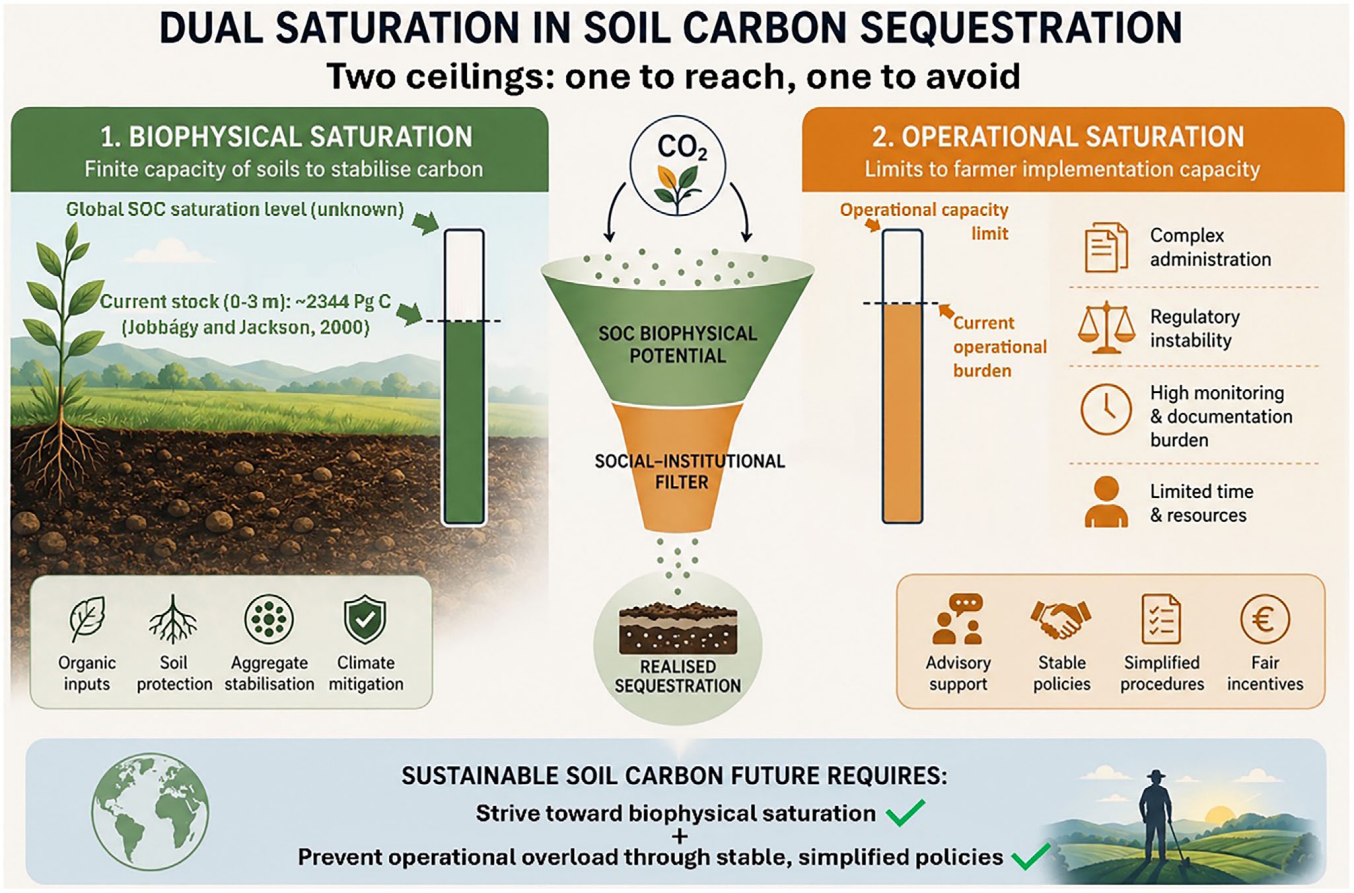


**Graphical abstract (corrected)**.

In paragraph 2 of the “The Biological Ceiling: The Knowledge Gap and Stoichiometric Constraints” section, the text “As Jobbágy and Jackson [5] showed, global soils currently store approximately 2344 Pg C down to a depth of 3 m, forming the largest terrestrial carbon reservoir directly influenced by land management decisions, although specific global estimates vary depending on depth and database used [23].” was incorrect. This should have read: “As Jobbágy and Jackson [5] showed, global soils have been estimated to contain approximately 2344 Pg C down to a depth of 3 m, forming the largest terrestrial organic carbon reservoir, substantial portions of which are influenced by land use and land management, although specific global estimates vary depending on depth and database used [23].”

Then, in paragraph 3 of the “The Biological Ceiling: The Knowledge Gap and Stoichiometric Constraints” section, the text “In contrast, managing a biological nitrogen source through the integration of specific legumes such as alfalfa (*Medicago sativa*), faba beans (*Vicia faba*), or clover‐grass leys to satisfy the C:N ratio defined by Tipping et al. [24] involves multi‐year planning and deep observational labor (Table 1).” was incorrect. This should have read: “In contrast, managing biological nitrogen supply through the integration of legumes such as alfalfa (*Medicago sativa*), faba bean (*Vicia faba*), or clover‐grass leys involves multi‐year planning and substantial observational and management effort (Table 1). Such practices may support the nutrient availability required for sustained SOC accumulation, although soil organic matter is not governed by a single universally applicable C:N ratio [24].”

In the penultimate paragraph of the “The Human Ceiling: Beyond Legislative Periods” section, the text: “Every hour spent navigating regulatory flux–the periodic adjustment of eligibility criteria, reporting requirements, and compliance rules across successive policy cycles within the European Common Agricultural Policy–is an hour diverted from field‐based observation [40]. When reporting requirements become dominant in farm management routines, time available for field observation and adaptive decision‐making may decrease, potentially reducing what has been described as farmers’ “situational awareness”[41].” was wrong. This should have read: “Time devoted to navigating changing eligibility criteria, reporting requirements, and compliance rules may reduce the time available for field observation and adaptive decision‐making. This is a conceptual inference from the documented administrative and advisory constraints rather than a directly quantified one‐for‐one relationship [40, 41].”

**TABLE 1** | The transition friction – mechanical vs. biological paradigms.

**Table:** 

Feature	Mechanical paradigm (industrial)	Biological paradigm (regenerative)
Primary nitrogen source	Synthetic fertilizer (e.g., urea application)	Leguminous fixation (e.g., clover ley, faba bean rotation) & organic matter
Predictability	High (direct linear yield response)	Variable (dependent on soil biology/weather)
Operational labor	Low (single tractor pass)	High (cover crop establishment, termination, monitoring)
Cognitive load	Low (standardized application rates)	High (site‐specific observational mastery)
Administrative burden	Low (minimal reporting)	High (complexity of “green” compliance)
Risk profile	Financial (input price volatility)	Operational (biological failure/harvest loss)

In the last paragraph of the “The Human Ceiling: Beyond Legislative Periods” section, the text “While SOC requires 20–50 years to reach a new equilibrium [42], the current administrative environment forces farmers into a reactive, short‐term compliance mode.” was wrong. It should have read: “While SOC requires decades to reach a new equilibrium (depending on soils, climate and management practices) [42], the current administrative environment forces farmers into a reactive, short‐term compliance mode.”

In the first paragraph of the “Policy as a ‘Tragedy of the Horizon’” section, the text “The physical and microbial timeframes required to reach a new SOC equilibrium are measured in decades (20–50 years) [42]” was incorrect. This should have read: “The physical and microbial timeframes required to approach a new SOC equilibrium are commonly measured in decades, depending on soils, climate, and management practices [42].”

Then, in the third paragraph of the “Policy as a ‘Tragedy of the Horizon’” section, the text “1. Long‐term regulatory horizons: agricultural policies must be decoupled from short‐term election cycles [45], providing farmers with 10‐to‐20‐year stability to realize the biological transitions necessary for SOC equilibrium.” was wrong. This should have read: “1. Long‐term regulatory horizons: agricultural policies must be decoupled from short‐term election cycles, providing farmers with 10–20 years of planning security to realize the biological transitions necessary for SOC accumulation and stabilization. Such long‐term horizons would also facilitate the establishment and maintenance of long‐term field experiments that are required to evaluate perennial and other slow‐developing agricultural systems beyond typical short‐term funding cycles [45].”

The author apologizes for these errors and for any confusion they may have caused.

